# The relevance of m^6^A RNA modifications in cancer stem cells

**DOI:** 10.1007/s00432-026-06525-6

**Published:** 2026-06-09

**Authors:** Alexandra Monteiro, Diana Pádua, Catarina Gonçalves, Patrícia Mesquita, Raquel Almeida

**Affiliations:** 1 Faculty of Medicine, University of Porto, 4200-319 Porto, Portugal; 2 i3S-Institute for Research and Innovation in Health, University of Porto, 4200-135 Porto, Portugal; 3 ICBAS-School of Medicine and Biomedical Sciences, University of Porto, 4050-313 Porto, Portugal; 4 Biology Department, Faculty of Sciences, University of Porto, 4169-007 Porto, Portugal

**Keywords:** m^6^A, epitranscriptomics, cancer stem cells, cellular plasticity, cancer metabolism, therapy resistance

## Abstract

Cancer is a multifactorial disease, driven by dysregulation of cellular processes that are controlled by the complex influence of DNA and RNA-level regulatory mechanisms. Epitranscriptomics has surfaced as a prominent field in cancer research, providing a clearer understanding of post-transcriptional regulation of gene expression. In particular, N^6^-methyladenosine (m^6^A) is the most abundant internal modification in eukaryotic mRNA, and has emerged as a key regulator of post-transcriptional gene expression. Through the coordinated activity of methyltransferases (“writers”), demethylases (“erasers”) and binding proteins (“readers”), m^6^A dynamically modulates important RNA processing steps, including translation, stability, decay and splicing. Notably, m^6^A regulatory proteins are implicated in several stemness pathways acting as oncogenic drivers or tumor suppressors in a highly context-dependent manner. This regulatory flexibility is particularly relevant in cancer stem cells (CSCs), a highly plastic subpopulation involved in tumor initiation, progression, metastasis and therapy resistance. Indeed, some of the major challenges in oncology arise from cancer cell adaptability, tumor heterogeneity and microenvironment-dependent outcomes, processes in which m^6^A emerges as a critical regulatory layer. This review summarizes the mechanisms of m^6^A regulation and its biological effects, and provides an overview of the influence of m^6^A in CSC phenotypes. Finally, it explores how m^6^A affects therapeutic responses, future perspectives in oncology and the key challenges currently under investigation in this field.

## Introduction

Understanding the pathological mechanisms underlying cancer remains a major scientific challenge. Cancer is a highly complex and multifactorial disease characterized by extensive molecular and cellular heterogeneity, which contributes to diverse clinical outcomes and variable therapeutic responses. Recent advances have shown that tumors consist of dynamic cellular populations capable of adapting to environmental and therapeutic pressures (Pérez-González et al. 2023). This cellular plasticity represents a hallmark of cancer, enabling malignant cells to transition between distinct phenotypic states and thereby promoting tumor progression, metastatic dissemination, and resistance to therapy. In particular, processes such as epithelial–mesenchymal transition (EMT) and the acquisition of stem cell-like properties have been strongly implicated in driving these adaptive behaviors (Pérez-González et al. 2023).

Elucidating the regulatory mechanisms that control cellular plasticity and stemness is therefore essential for the development of more effective and durable anticancer therapies (Pérez-González et al. 2023). It has been increasingly recognized that cancer stem cells (CSCs) play a major role in tumor initiation, progression, relapse and therapy resistance; however, despite their clinical relevance, a consensus definition has yet to be established. The main attributes of CSCs include self-renewal capacity (Lee et al. 2025), differentiation into heterogeneous tumor cell populations (Pérez-González et al. 2023) and remarkable adaptability, allowing survival under environmental stress and evasion of treatment approaches (Pan et al. 2025). The identification of CSCs is further complicated by the absence of universal markers, as many surface proteins are shared with non-tumorigenic stem cells and their expression varies across tumor types. Additionally, stem-like properties can be acquired *de novo* by non-CSCs under adverse conditions such as hypoxia, inflammation or therapeutic pressure, supporting the concept of CSCs as a flexible functional state rather than a rigid subpopulation (Lee et al. 2025).

The dynamic plasticity observed in CSCs is regulated by multiple layers of gene expression control. Increasing evidence highlights that although genetic mutations play a central role, they are not the only drivers of tumor development and cannot fully explain the adaptive behavior observed in cancer cells (Yue et al. 2023). Epigenetic mechanisms, including DNA methylation and histone modifications, contribute significantly to the regulation of cellular states and tumor progression (Feinberg and Levchenko 2023). Furthermore, emerging studies show that chemical modifications of RNA molecules, currently referred to as epitranscriptomics, as well as proteomic heterogeneity, are key contributors to shaping this cellular identity (Chua et al. 2020). Among these modifications, N^6^-methyladenosine (m^6^A), the methylation of adenosines at the nitrogen-6 position, is the most prevalent and conserved internal RNA modification in eukaryotic cells (Jiang et al. 2021) and has gained particular attention due to its dynamic and reversible nature, as well as its role in cancer progression and stemness regulation (Shi et al. 2022).

One important consideration is that m^6^A regulators do not consistently act as purely oncogenic or tumor-suppressive factors. Rather, their roles are highly context-dependent, being influenced by the cellular environment, cancer type and developmental stage. In this context, m^6^A-modifying proteins regulate core stemness networks, thereby controlling processes such as self-renewal and differentiation, which are critical for neoplastic progression (Liang et al. 2020).

As evidence continues to grow regarding the impact of m^6^A in CSC biology, understanding its molecular and functional roles has become increasingly important. This review provides an overview of current knowledge on the mechanisms regulating m^6^A and their contribution to CSC characteristics. We first explore the molecular machinery responsible for m^6^A deposition, removal and recognition. Next, we discuss how m^6^A regulation affects key CSC traits, including self-renewal, pluripotency, tumor initiation and metastization capacity, plasticity, immune evasion, metabolic reprogramming and therapy resistance. Finally, we explore the emerging clinical applications, potential therapeutic strategies and the challenges associated with targeting m^6^A-mediated processes in oncology.

## Biological functions of m^6^A

Recent advances in RNA biology have identified approximately 170 distinct RNA modifications, distributed across messenger RNA (mRNA), transfer RNA (tRNA), ribosomal RNA (rRNA), lncRNA (long noncoding RNA), microRNAs (miRNAs), small nuclear RNA (snRNA) and small nucleolar RNA (snoRNA) (Huang et al. 2020; Höffler and Duss 2023). While most RNA modifications are considered static, m^6^A is among the few shown to be reversible (Boo and Kim 2020). The addition of this mark is catalyzed by methyltransferase complexes (“writers”), whereas its removal is mediated by demethylases (“erasers”) (Esteve-Puig et al. 2020; Chua et al. 2020). A third group of proteins, termed “readers”, specifically recognizes this modification, thereby mediating its biological functions (Huang et al. 2020). Through the regulation of post-transcriptional processes, m^6^A modulates RNA metabolism, including mRNA stability, translation, decay, export, splicing, folding, editing and processing (Liao et al. 2022; An and Duan 2022). Given its central role in RNA metabolism, m^6^A represents a key regulatory layer with significant potential to advance cancer research and inform the development of more effective therapeutic strategies (An and Duan 2022). Emerging evidence highlights that m^6^A methylation plays a fundamental role in regulating gene expression through RNA-level modifications, particularly in contexts demanding rapid cellular remodeling, such as adverse conditions and intracellular stress (Wang et al. 2021; Hong et al. 2022). This may create conditions that favor malignant transformation and disease progression.

Aberrant regulation of m^6^A “writers”, “erasers” and “readers” can lead to improper processing of RNA transcripts encoding key oncogenes, stemness factors and tumor suppressors. Depending on the cancer type, cellular context and environmental conditions, these transcripts can be either overexpressed or repressed, leading to distinct biological outcomes, as discussed in the following sections (Hong et al. 2022).

## Overview of m^6^A machinery

### “Writers”

**Fig. 1:**
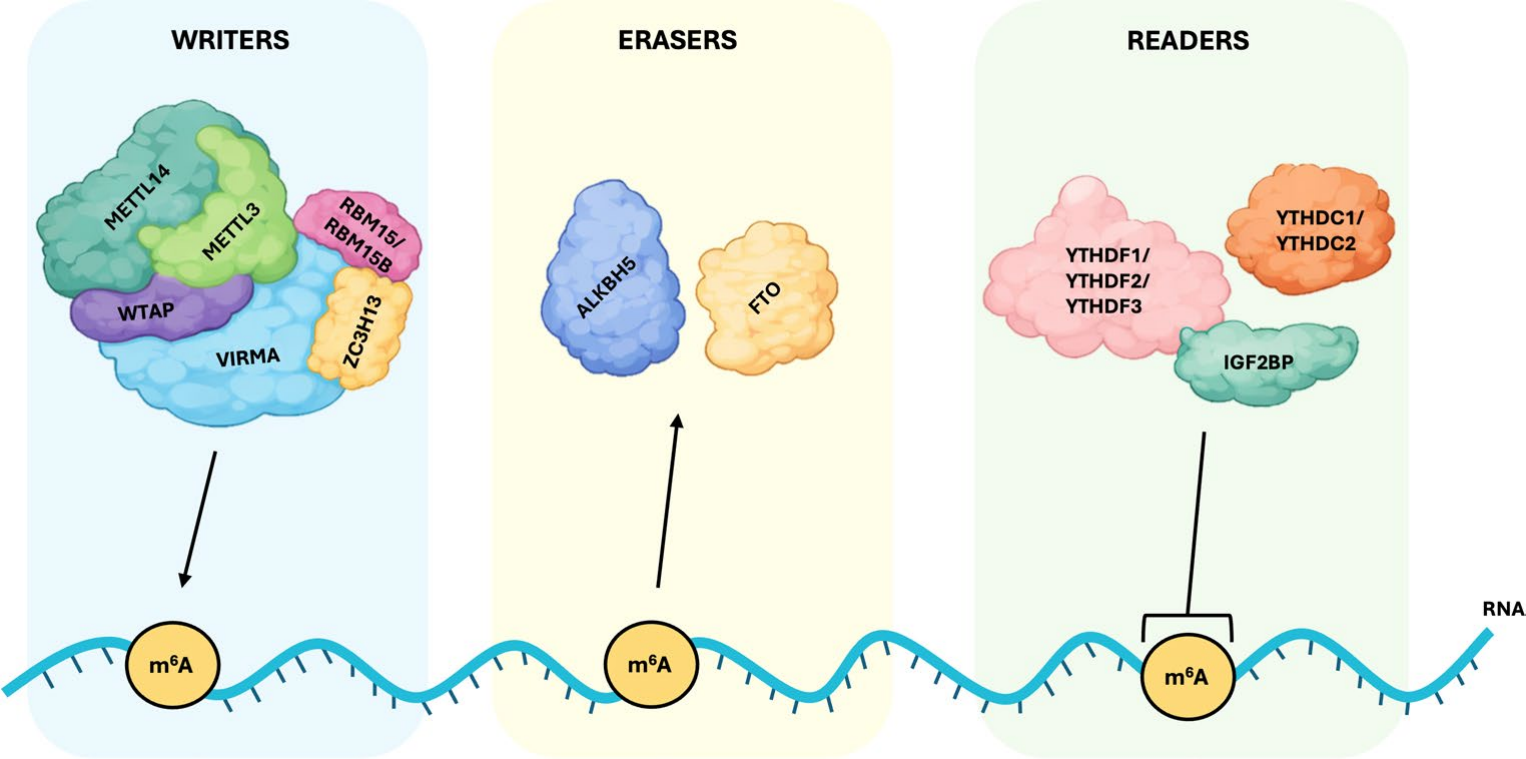
m^6^A RNA modification machinery. Schematic representation of the dynamic regulation of N6-methyladenosine (m^6^A) on RNA, including “writers” (METTL3, METTL14, WTAP, VIRMA, ZC3H13, RBM15/RBM15B), “erasers” (ALKBH5, FTO) and “readers” (YTHDF1/YTHDF2/YTHDF3, YTHDC1/2, IGF2BP), which modulate RNA processes such as translation, stability, decay and splicing

The deposition of m^6^A modifications on RNA molecules is mediated by a multi-component methyltransferase complex (Fig. 1). The core m^6^A “writer” complex consists of METTL3 (methyltransferase-like protein 3), METTL14 (methyltransferase-like protein 14), WTAP (Wilms tumor 1-associated protein), VIRMA (KIAA1429, vir-like m^6^A methyltransferase associated protein), RBM15 and its paralog RBM15B (RNA binding motif proteins 15 and 15B, respectively) and ZC3H13 (zinc finger CCCH-type containing 13 protein) (Haruehanroengra et al. 2020; Kim and Lee 2021). Additional methyltransferases, such as METTL16 and the cap-specific enzyme PCIF1, have also been described, although they typically target specific RNA subsets or related modifications (Warda et al. 2017; Zaccara et al. 2019; Sendinc et al. 2019). METTL3 is the catalytic subunit responsible for transferring the methyl group to specific sites onto the RNA molecule. However, several accessory proteins further stabilize and guide the complex (Fig. 1). METTL14, although catalytically inactive, forms a stable heterodimer with METTL3 and acts as an RNA-binding scaffold and allosteric activator (Wang et al. 2016). WTAP functions as a regulatory subunit that recruits the METTL3-METTL14 complex to target transcripts, particularly within nuclear speckles (Śledź and Jinek 2016; Zaccara et al. 2019; Huang et al. 2022). VIRMA, the largest component of the m^6^A “writer” complex, acts as a scaffold that facilitates the association of the METTL3-METTL14 core with regulatory proteins (WTAP, RBM15/RBM15B and ZC3H13) and the RNA substrate (Lu et al. 2025b). RBM15/RBM15B function as mRNA-binding adaptors that recruit the complex to specific sites, often recognizing uridine-rich regions, while ZC3H13 contributes to nuclear localization and structural integrity of the complex, ensuring efficient methylation (Shen et al. 2025).

### “Erasers”

The “erasers” are demethylases that remove the m^6^A modification, contributing to its reversible nature (Fig. 1). Currently, two major “erasers” have been identified (Fig. 1), FTO (Fat Mass and Obesity-associated protein) and ALKBH5 (α-ketoglutarate-dependent dioxygenase alkB homolog 5), both members of the AlkB family (Esteve-Puig et al. 2020; Gao et al. 2024; Jia et al. 2011). FTO and ALKBH5 share a double-stranded β-helix fold that underlies their RNA demethylase activity, along with additional domains that can modulate enzymatic function. Despite their common role in removing m^6^A, they exhibit notable structural differences, particularly in their secondary structure (Gao et al. 2024). In addition, FTO demethylates both internal m^6^A and cap-adjacent m^6^Am (N^6^,2’-O-dimethyladenosine), which is exclusively found at the first nucleotide following the 7-methylguanosine cap in many mRNAs and decreases the stability of m^6^Am-containing transcripts by inducing RNA decay. This evidence hints that FTO may act on various substrates (Yang et al. 2018; Mauer et al. 2017). ALKBH5, in contrast, is primarily a nuclear m^6^A demethylase involved in RNA processing and nuclear export, showing selectivity for m^6^A residues within specific sequence motifs, rather than broadly targeting methylated bases present in single-stranded RNA (Yang et al. 2018).

### “Readers”

The biological functions of m^6^A are mediated by “reader” proteins that selectively bind transcripts containing this modification (Fig. 1). Several families of “readers” have been described, including YTHDF proteins (YTH domain-containing family: YTHDF1, YTHDF2, YTHDF3); YTHDC proteins (YTH domain-containing family: YTHDC1 and YTHDC2) and IGF2BP (Insulin-like growth factor 2 mRNA-binding proteins) (Zaccara et al. 2019; Esteve-Puig et al. 2020).

YTHDF1 enhances the translation of m^6^A-modified mRNA, whereas YTHDF2 recruits RNA decay machinery to target transcripts. In turn, YTHDF3 modulates the activity of both YTHDF1 and YTHDF2 activities (Sikorski et al. 2023). Unlike YTHDF1-3, which are primarily cytoplasmic, YTHDC1 is nuclear and regulates RNA splicing, promoting exon inclusion and mRNA export to the cytoplasm (Zhen et al. 2020). YTHDC2, a cytoplasmic protein with a helicase domain, regulates mRNA stability, decay, and translation, and can also play roles in meiosis, with functions that vary depending on the cellular context (Xu et al. 2021). Finally, IGF2BP proteins modulate RNA stability, translation and localization, affecting multiple biological processes (Duan et al. 2024).

## m^6^A regulation of core CSC phenotypes

**Fig. 2:**
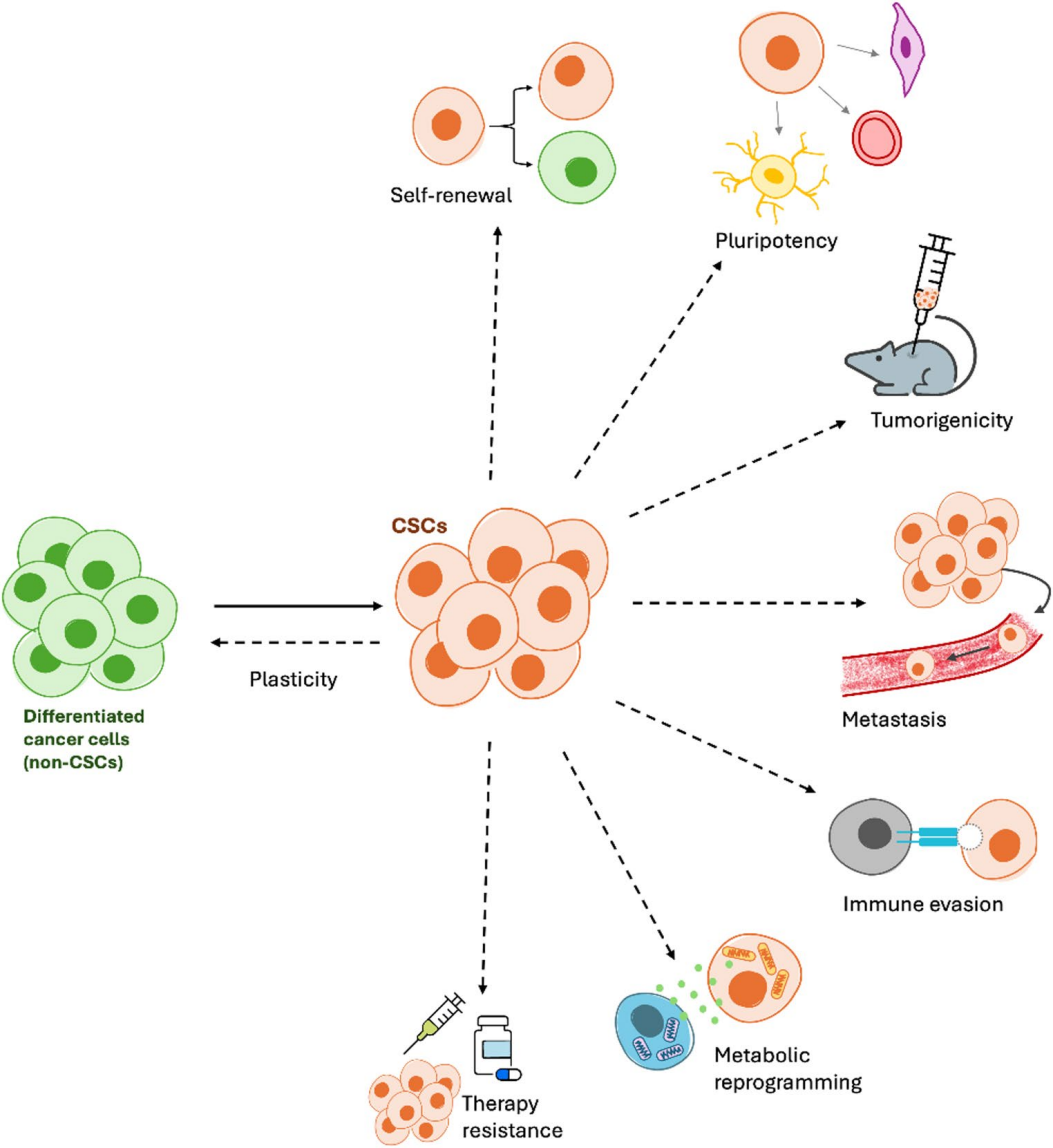
Functional roles of CSCs. Diagram illustrating the central role of CSCs in tumor progression. Key CSC properties, such as pluripotency, self-renewal, and immune evasion, facilitate tumorigenic processes, leading to increased metastatic potential, long-term tumor-propagating capacity, intratumoral heterogeneity, and resistance to conventional therapies, including chemotherapy, radiotherapy and immunotherapy

CSCs have been identified across multiple tumor types, including hematologic malignancies and solid cancers (Batlle and Clevers 2017). Several tissues, including epithelial and hematopoietic tissues, sustain continuous self-renewal through the activity of multipotent stem cells residing in specialized niches. These cells possess the ability both to maintain their own population and to differentiate into the diverse cell types that constitute each tissue. In cancer, a similar process is driven by CSCs, which generate heterogeneous tumors organized in a hierarchical manner. CSCs are characterized by stem-like properties, including pluripotency and self-renewal capacity, which contribute to tumor initiation, metastasis and resistance to therapy (Zhang et al. 2024; Chu et al. 2024). These features (Fig. 2) underscore the critical role of CSCs in disease progression and highlight them as promising targets for future oncologic therapies.

Several studies have demonstrated that m^6^A RNA modifications and their associated regulatory machinery play critical roles in modulating CSC behavior, as discussed in the following sections and highlighted in Table 1. Understanding the biological mechanisms underlying these processes is essential for clarifying how m^6^A RNA modifications contribute to the CSC phenotype and drive cancer development and progression.

### Self-renewal and pluripotency

Self-renewal is the capacity of CSCs to divide asymmetrically, originating one differentiated cell and one cell that preserves pluripotent properties (Krause and Van Etten 2007). This process is closely regulated by m^6^A, through the stabilization of transcripts encoding key pluripotency factors such as NANOG, SOX2 and OCT4 (Yang et al. 2026). In cancer, this process has also been shown to be modulated by m^6^A methylation of key stemness transcripts in different cancer models. METTL3, together with METTL14, is frequently overexpressed in several cancer types and contributes to stem cell maintenance, dedifferentiation and cell growth, acting as an oncogenic driver in glioblastoma, bladder and colorectal cancer (Cui et al. 2017; Gao et al. 2020; Sun et al. 2021; Pádua et al. 2025).

**Table 1:** m^6^A regulators in CSC biology and therapeutic targeting

m^6^A regulator	Cancer type	CSC-related phenotypes	Mechanism	References
METTL3	Glioblastoma	Stemness and pluripotency	SOX2 mRNA stabilization	Visvanathan et al. (2018)
		Stemness and invasiveness	BRAFV600E/MEK/ERK/METTL3	Xie et al. (2025)
	Bladder	Self-renewal, tumorigenicity	AFF4-SOX2/MYC	Gao et al. (2020)
	Colorectal	Stemness, tumorigenicity and metastasis	IGF2BP2-SOX2	Li et al. (2019)
		Immune evasion	IFN-γ-Stat1-Irf1	Wang et al. (2020)
	Pancreas	Chemo- and radioresistance	MAPK	Taketo et al. (2018)
METTL14	Bladder	Self-renewal, tumorigenicity, and metastasis	NOTCH1	Gu et al. (2019)
	Colorectal	Immune evasion	IFN-γ-Stat1-Irf1	Wang et al. (2020)
	Lung	Stemness, tumorigenicity, metabolic reprogramming, chemoresistance	GPX2-GLI-SHH	Yang et al. (2025)
WTAP	Ovary	Metabolic reprogramming, tumorigenicity and therapy resistance	WTAP-DGCR8-miR-200-HK2	Lyu et al. (2022)
	Gastric	Migration, chemotherapy; chemo- and radio-resistance	TGF-β induced EMT	Liu and Da (2023)
FTO	Bladder	Pluripotency and self-renewal	CD133, CD44, NANOG, and OCT4; EMT	Wu and He (2026)
	Leukemia	Self-renewal and immune evasion	immune checkpoint genes, especially LILRB4	Su et al. (2020)
		Stemness-associated markers, tumorigenicity and chemoresistance	Targets CD133-expressing cells	Bian et al. (2025)
ALKBH5	Breast	Pluripotency	NANOG stabilization	Zhang et al. (2016)
		Metabolic reprogramming and chemoresistance	ALKBH5/GLUT4	Liu et al. (2022)
	Glioblastoma	Self-renewal	LATS2	Wei et al. (2025)
		Self-renewal, metabolic reprogramming and therapy resistance	MAS-MDH2-α-KG-ALKBH5	Lv et al. (2024)
	Colorectal	CSC marker expression, tumorigenicity, chemoresistance	FAM84A-β-catenin	Zhou et al. (2025)
		Migration, metabolic reprogramming and immune evasion	AXIN 2- Wnt-β-catenin; PD-L1	Zhai et al. (2023)
YTHDF1	Colorectal	Tumorigenicity	Wnt/β-catenin	Bai et al. (2019)
		Immune evasion	p65-CXCL1; PD-1	Bao et al. (2023)
	Breast	Invasion, tumorigenicity, metastasis and metabolic reprogramming	HIF1α-miR-16-5p-YTHDF1-PKM2	Yao et al. (2022b)
YTHDF2	Lung	Tumorigenicity, metastasis	AXIN1-Wnt-β-catenin	Li et al. (2021)
	Colorectal	Immune evasion	PD-1/PDL-1	Xiao et al. (2024)
	Ovary	Immune evasion	PD-1/PDL-1	Xiao et al. (2024)
YTHDC1	Leukemia	Self-renewal	Alternative splicing of HOXB-AS3	Wu et al. (2023)
	Head and neck	Stemness	SOX2 and BMI1	Weng et al. (2022)
YTHDC2	Bladder	Invasion, migration and self-renewal	SOX2	Cai et al. (2025a)
IGF2BP1	Gastric	Immune evasion	PD-L1	Tang et al. (2024)
IGF2BP3	Naso-pharyngeal	Metastasis, tumorigenicity and stemness	NOTCH3	Chen et al. (2023a, b)
	Bladder	Stemness and immune evasion	SPHK1; PDL-1	Wang et al. (2024)
	Leukemia	Metabolic reprogramming	Serine synthesis pathway (SSP) genes (ATF4, PHGDH, PSAT1)	Huang et al. (2025)
	Breast (TNBC)	Stemness, tumorigenicity and chemoresistance	FZD1/7-β-catenin	Cai et al. (2025b)

AFF4 - ALF transcription elongation factor 4; AS3 - PDS5 cohesin associated factor B; ATF4 - activating transcription factor 4; AXIN1- axin 1; AXIN2 - axin 2; BMI1 - BMI1 proto-oncogene, polycomb ring finger; BRAF - B-Raf proto-oncogene; CD133 - prominin 1; CD44 – CD44 molecule; CXCL1 - C-X-C motif chemokine ligand 1; DGCR8 - DGCR8 microprocessor complex subunit; EMT - Epithelial-Mesenchymal Transition; ERK - mitogen-activated protein kinase 1; FAM84A - LRAT domain containing 1; FZD1/7 - frizzled class receptor 1/7; GLI - GLI family zinc finger 1; GLUT4 - solute carrier family 2 member 4; GPX2 - glutathione peroxidase 2; HIF1α - hypoxia inducible factor 1 subunit alpha; HK2 - hexokinase 2; HOXB - homeobox B cluster; IFN-γ - Interferon gamma; Irf1 - interferon regulatory factor 1; LATS2 - large tumor suppressor kinase 2; LILRB4 - leukocyte immunoglobulin like receptor B4; MAPK-mitogen activated kinase-like proteinMAS - MAS1 proto-oncogene, G protein-coupled receptor; MDH2 - malate dehydrogenase 2; MEK - mitogen-activated protein kinase kinase 7; miR - micro RNA; MYC - MYC proto-oncogene, bHLH transcription factor; NANOG - Nanog homeobox; NOTCH1 - notch receptor 1; NOTCH3 - notch receptor 3; OCT4 - POU class 5 homeobox 1; p65 - RELA proto-oncogene, NF-kB subunit; PD-1 - RPL17-C18orf32 readthrough; PD-L1 - CD274 molecule; PHGDH - phosphoglycerate dehydrogenase; PKM2 - pyruvate kinase M1/2; PSAT1 - phosphoserine aminotransferase 1; SHH - Sonic hedgehog; SOX2 - SRY-box transcription factor 2; SPHK1 - phingosine kinase 1; SSP - Serine synthesis pathway; Stat1 - signal transducer and activator of transcription 1; TGF-β - transforming growth factor beta 1; V600E - specific mutation where valine is replaced by glutamic acid at position 600 of BRAF; Wnt - Wnt signaling pathway; α-KG - alpha-ketoglutarate; β-catenin - catenin beta 1

In glioma stem-like cells, the BRAF V600E mutation markedly upregulates METTL3 expression through activation of ERK signaling, thereby enhancing stemness and invasiveness (Xie et al. 2025). Additional m^6^A regulators also contribute to CSC maintenance; for instance, the demethylase ALKBH5 regulates NANOG expression, while m^6^A “reader” proteins, such as IGF2BPs, stabilize transcripts encoding key pluripotency factors, including SOX2 and OCT4 (Yang et al. 2026). On the other hand, in colorectal cancer, ALKBH5 expression positively correlates with CSC markers and promotes self-renewal as well as the expression of stemness-associated genes in CSCs and patient-derived organoids. Consistently, tumorigenesis is significantly reduced in colon stem cell-specific Alkbh5 knockout mice (Zhou et al. 2025). Similarly, in bladder cancer, transcriptomic analyses of FTO-high patient samples and FTO-knockdown cells revealed a strong association between FTO expression and activation of EMT- and CSC-related pathways. Accordingly, FTO silencing impaired the self-renewal capacity of bladder cancer cells and reduced the expression of key stemness markers, including CD133, CD44, NANOG, and OCT4 (Wu and He 2026). A role for YTHDC1 in the regulation of leukemia stem cell self-renewal in acute myeloid leukemia has also been demonstrated (Cheng et al. 2021; Sheng et al. 2021; Wu et al. 2023). YTHDC1 expression increased the number of leukemia stem cells in the blood and bone marrow of acute myeloid leukemia mouse models (Wu et al. 2023). YTHDC1 has also been implicated in the maintenance of CSCs in solid tumors, including head and neck squamous cell carcinoma (Weng et al. 2022).

In addition to directly regulating pluripotency factors, m^6^A can also shape key stemness-associated signaling pathways, including MAPK, NF-κB, Wnt, PI3K/AKT and mTOR, which govern CSC lineage commitment, proliferation and apoptosis (Xie et al. 2025; Wei et al. 2025, Xu et al. 2026). Moreover, m^6^A-mediated regulation affects tumor suppressors such as TP53, PTEN, CDKN2A and LATS2, which have key functions in cell cycle regulation and differentiation. For example, phosphorylated ALKBH5 can demethylate LATS2 mRNA, increasing its stability by preventing YTHDF2-mediated decay. Knockdown of LATS2 has been shown to reduce the self-renewal capacity of glioblastoma stem cells, highlighting a specific m^6^A-dependent axis critical for stemness (Wei et al. 2025).

In light of these findings, dysregulated activity of m^6^A “writers,” “erasers,” and “readers” can lead to aberrant expression of pluripotency-associated factors, thereby promoting CSC proliferation and contributing to tumor progression (Cui et al. 2017; Gao et al. 2020; Sun et al. 2021; Pádua et al. 2025). However, the functions of the m^6^A machinery are not uniform and appear to be highly context-dependent. Rather than acting independently, the coordinated interplay among multiple m^6^A regulators likely determines the ultimate biological outcome of these processes (Yang et al. 2026).

### Tumorigenicity and metastasis

Beyond sustaining self-renewal and pluripotency, the m^6^A machinery can critically influence the tumor-initiating and metastatic capacities of CSCs (Table 1). Tumorigenicity reflects the ability of a cell or a population of cells to reconstitute a tumor, a process strongly associated with stemness-related transcriptional programs (Ben-David and Benvenisty 2011). For instance, in colorectal cancer, silencing of YTHDF1 suppresses Wnt/β-catenin signaling (Bai et al. 201). Similarly, YTHDF2 is upregulated in lung adenocarcinoma and facilitates tumorigenesis and metastasis through m^6^A-dependent regulation of the AXIN1/Wnt/β-catenin signaling pathway, which is associated with the maintenance of CSCs (Li et al. 2021). In glioma CSCs, METTL3 is highly expressed, with levels progressively declining as cells undergo lineage commitment (Visvanathan et al. 2018). IGF2BP3, a member of the IGF2BP reader family, is highly expressed in nasopharyngeal carcinoma, particularly in metastatic cases, and correlates with poor prognosis (Chen et al. 2023a). Mechanistically, IGF2BP3 induces NOTCH3 signaling through m^6^A-dependent stabilization of NOTCH3 mRNA, sustaining pathway activation and driving the expression of stemness-related genes. This contributes to metastasis by increasing the tumor-initiating capacity of disseminated tumor cells in vivo (Chen et al. 2023b). These findings can be corroborated by those from Radhi et al. that observed increased expression of METTL3, WTAP and FTO transcripts and proteins as well as m^6^A-modified RNAs in cells derived from invasive regions of glioblastoma, while elevated WTAP and FTO expression significantly correlated with poor patient survival (Radhi et al. 2026). Moreover, functional depletion of these proteins significantly altered m^6^A levels and impaired self-renewal and cell invasion behavior. Notably, in bladder cancer, m^6^A seems to have the opposite effect, as depletion of YTHDC2 significantly increased the pool of bladder CSCs resulting in a phenotypic shift towards a more invasive subtype of bladder cancer. This shift was characterized by enhanced proliferation, migration, invasion, and self-renewal capabilities of cancer cells, highlighting YTHDC2’s function as a tumor suppressor (Cai et al. 2025a). Also, in this cancer type, METTL14 expression is reduced, and its loss enhances metastasis and self-renewal through NOTCH1 m^6^A methylation (Gu et al. 2019; Wei et al. 2025).

Collectively, these findings illustrate how m^6^A-mediated regulation reinforces CSC properties, thereby promoting metastatic potential and disease progression. Notably, the functional impact of m^6^A is highly context-dependent as in some cancers, its regulators act as oncogenic drivers, whereas in others they exert tumor-suppressive effects (Yuan et al. 2025).

### Plasticity and immune evasion

The tumor immune microenvironment (TIME) plays a central role in shaping CSC plasticity and promoting immune evasion (Batlle and Clevers 2017; Pan et al. 2025). Through cytokines, chemokines, and extracellular vesicles, tumor and immune cells establish immunosuppressive niches that support CSC maintenance, epithelial–mesenchymal transition (EMT), metastasis and therapy resistance (Pan et al. 2025; Shibue and Weinberg 2017; Liu et al. 2023; Meacham and Morrison 2013). CSCs evade immune surveillance through upregulation of immune checkpoint molecules such as PD-L1, modulation of antigen-presentation and epigenetic and epitranscriptomic reprogramming, while also recruiting regulatory T cells (Tregs), myeloid-derived suppressor cells (MDSCs), and tumor-associated macrophages (TAMs) to reinforce immune suppression (Pan et al. 2025). m^6^A RNA modification has emerged as a key regulator of the CSC-TIME interplay, controlling CSC plasticity and contributing to immune adaptation (Zhang et al. 2021). Emerging evidence suggests that m^6^A regulators may influence responses to immunotherapy by modulating the immunological state of the TIME. Aberrant m^6^A signaling contributes to immune escape by modulating immune checkpoint expression, cytokine signaling, and inflammatory pathways (Lu et al. 2025a; Wang et al. 2020; Han et al. 2019). Elevated expression of METTL3, METTL14, YTHDF1/2, and IGF2BP1/3 has been linked to increased PD-L1 stability and suppression of antitumor T-cell activity in several malignancies (Han et al. 2019; Wang et al. 2020, 2024; Bao et al. 2023; Xiao et al. 2024; Tang et al. 2024). In particular, m^6^A demethylases such as ALKBH5 and FTO regulate cytokine signaling, hypoxia response and immune cell infiltration, thereby supporting CSC maintenance and tumor progression (Zhai et al. 2023; Li et al. 2026). Collectively, these findings position the m^6^A–CSC axis as a key interface linking stemness, cell plasticity, immune evasion and therapeutic resistance across cancer types.

Recent advances in single-cell and spatial epitranscriptomics are providing unprecedented insights into the heterogeneity of cancer cells and their interactions with the TIME (Ståhl et al. 2016, Longo et al, 2021). Emerging technologies such as scMeRIP-seq and scDART-seq, and spatial epitranscriptomics enable the analysis of m^6^A modifications at the individual cell level, while preserving tissue architecture and microenvironmental context (Dierks et al. 2021; Yao et al. 2022a; Tegowski et al. 2022; Bao et al. 2023). By integrating spatial and epitranscriptomic data, it becomes possible to identify CSC niches characterized by distinct m^6^A-dependent regulatory programs associated with immunotherapeutic response (Wang et al. 2024; Mao et al. 2026). Importantly, these approaches are expanding the translational potential of m^6^A research by identifying patient-specific epitranscriptomic vulnerabilities, ultimately guiding CSC-targeted therapeutic strategies, including inhibitors of METTL3, FTO, and ALKBH5 (Su et al. 2020; Yankova et al. 2021; Mao et al. 2026).

### Metabolic reprogramming

Another mechanism through which m^6^A modifications influence CSC biology is the regulation of cellular metabolism (Yue et al. 2023). Increasing evidence indicates that m^6^A regulators modulate glycolytic pathways across multiple cancer types, frequently promoting the Warburg effect, whereby cancer cells preferentially utilize glycolysis over oxidative phosphorylation, even under normoxic conditions (Peiris-Pagès et al. 2016; Yue et al. 2023). This metabolic reprogramming facilitates adaptation to hostile environments, such as hypoxia, while increased lactate production contributes to immune evasion and tumor progression (Yue et al. 2023). Several studies support the role of m^6^A regulators in tumor metabolic adaptation. For example, in ovarian cancer, HIF-1α regulated the m^6^A writer WTAP promoting the Warburg effect of ovarian cancer by m^6^A-dependent manner (Lyu et al. 2022). Expression of WTAP is significantly increased in high-grade tumors and in cases with lymph node metastasis, while elevated WTAP levels are associated with poorer patient survival independently of tumor stage or subtype, suggesting potential prognostic value (Lyu et al. 2022). Mechanistically, WTAP depletion suppresses glycolysis and enhances oxidative phosphorylation-dependent ATP production, highlighting its contribution to metabolic reprogramming (Lyu et al. 2022). Hypoxia-induced epitranscriptomic remodeling further reinforces CSC maintenance. Under hypoxic conditions, HIF-1α and HIF-2α upregulate the m^6^A demethylase ALKBH5 in breast cancer cells, promoting CSC pluripotency through stabilization of NANOG mRNA (Zhang et al. 2016). YTHDF1 facilitates proliferation and invasion of breast cancer cells and enhances tumorigenicity and metastasis through promoting glycolysis (Yao et al. 2022b). Glioblastoma stem cells also undergo metabolic adaptations that reshape their epitranscriptomic landscape. Specifically, these cells activate the malate–aspartate shuttle, leading to reduced levels of α-ketoglutarate, an essential cofactor for the demethylase ALKBH5, thereby sustaining tumor stemness programs. Importantly, pharmacological targeting of this metabolic pathway sensitized highly therapy-resistant cancer cells to targeted treatment (Lv et al. 2024). Similarly, a CRISPR/Cas9 screen identified IGF2BP3 and METTL14 as critical mediators of the metabolic vulnerability of acute myeloid leukemia (AML) cells and CSCs to serine and glycine deprivation. Depletion of either factor sensitized leukemia cells to nutrient restriction, demonstrating a key role for m^6^A signaling in serine metabolism. Moreover, IGF2BP3 silencing combined with dietary serine and glycine restriction suppressed AML progression both in vitro and in vivo while sparing normal hematopoiesis (Huang et al. 2025).

Although accumulating evidence supports a role for m^6^A-mediated metabolic regulation in CSC biology, many in vitro studies still rely on hyperoxic and high-glucose culture conditions that may not accurately reflect physiological tumor metabolism. Therefore, more physiologically relevant experimental models are required to clarify the contribution of epitranscriptomic metabolic reprogramming to CSC maintenance and therapeutic resistance (Peiris-Pagès et al. 2016).

### Therapy resistance

Resistance to cancer therapy is a major driver of treatment failure in clinical oncology. Understanding the underlying mechanisms is essential for the development of innovative and more effective therapeutic strategies. m^6^A modifications can influence treatment response by regulating processes such as metabolism, autophagy, DNA repair, drug metabolism and CSC activity (Shriwas et al. 2021). For instance, METTL3 upregulation is associated with resistance to radiotherapy and chemotherapeutic agents such as 5-fluorouracil, gemcitabine and cisplatin in pancreatic cancer (Taketo et al. 2018). In gastric cancer, WTAP has been reported to increase resistance to cyclophosphamide, cisplatin and radiotherapy (Liu and Da 2023). ALKBH5 overexpression promotes chemoresistance in colorectal cancer by enhancing stem-like properties in CSCs, patient-derived organoids, and transgenic mouse models. Conversely, genetic knockout or siRNA-mediated silencing of ALKBH5 synergizes with chemotherapy to induce significant tumor regression (Zhou et al. 2025). In triple-negative breast cancer, Cai et al. identified IGF2BP3 as a dominant m^6^A “reader” enriched in CSC populations through transcriptomic analyses (Cai et al. 2025b). Functional studies demonstrated that IGF2BP3 knockdown impaired stem-like properties and sensitized CSCs to carboplatin treatment, highlighting its role in therapy resistance. In non-small cell lung cancer, glutathione peroxidase 2 (GPX2) promotes CSC characteristics and resistance to tyrosine kinase inhibitors through activation of Hedgehog signaling. Notably, METTL14-mediated m^6^A modification of GPX2 mRNA decreases its stability, suggesting a regulatory mechanism through which m^6^A signaling modulates therapeutic resistance (Yang et al. 2025). Similarly, in leukemia, expression of the demethylase FTO and stemness-associated markers, including CD44, CD133, and CD25, is markedly elevated in tyrosine kinase inhibitor-resistant cells compared with parental cell lines. Importantly, pharmacological inhibition of FTO suppresses the growth of resistant leukemia cells, supporting its potential as a therapeutic target (Bian et al. 2025).

Emerging evidence further indicates that anticancer therapies themselves can reshape m^6^A methylation patterns, potentially contributing to treatment-associated toxicity and limiting clinical applicability. For example, the tyrosine kinase inhibitor sunitinib represses the m^6^A “eraser” FTO while upregulating METTL14 and WTAP, resulting in increased global m^6^A levels that correlate with cellular damage and contribute to cardiotoxicity in human-induced pluripotent stem cell-derived cardiomyocytes (Ma et al. 2022). Likewise, doxorubicin treatment increases m^6^A methylation in breast cancer cells, which is associated with enhanced DNA damage responses (Wu et al. 2022; Zhuang et al. 2023).

Overall, CSCs exploit m^6^A-mediated pathways to survive therapy, adapt metabolically, and maintain plasticity, contributing to tumor recurrence and poor prognosis yet advanced approaches are improving the detection and targeting of CSC-specific vulnerabilities, namely m^6^A modifications, offering hope for future precision therapies (Lee et al. 2025).

## Clinical implications

With the growing recognition of the diverse and critical roles of m^6^A in CSC biology, increased attention has focused on how these epitranscriptomic regulators may inform clinical decision-making. Dysregulated expression of m^6^A “writers”, “erasers” and “readers” has been consistently associated with cancer severity, patient prognosis and therapeutic response across multiple tumor types (Chai et al. 2019; Zheng et al. 2021; Xu et al. 2024). Consequently, these factors are emerging as promising noninvasive biomarkers for early cancer diagnosis, risk stratification and disease monitoring. For example, elevated m^6^A levels in peripheral blood leukocytes have been reported in lung squamous cell carcinoma compared with lung adenocarcinoma, exhibiting high sensitivity and specificity for distinguishing these cancer subtypes, and potentially improving clinical outcomes through more accurate diagnosis (Pei et al. 2020). In line with this, dysregulated METTL3, METTL14, and ALKBH5 expression has been linked to clinical progression and prognosis in gastric and esophageal cancer, while distinct m^6^A expression signatures correlate with patient survival and clinicopathological characteristics in colorectal adenocarcinoma (Li et al. 2019; Su et al. 2022). In hepatocellular carcinoma, m^6^A regulator profiles correlate with clinicopathological features, therapeutical and survival outcomes, further supporting their diagnostic, prognostic and predictive utility (Yan et al. 2024). Elevated IGF2BP3 expression also predicts poorer overall survival in acute myeloid leukemia and other malignancies (Zhang et al. 2022; Chen et al. 2023b). Collectively, these findings underscore the broad clinical potential of m^6^A regulators as biomarkers across diverse cancer types.

Beyond biomarker development, RNA-editing technologies such as CRISPR-Cas13 systems, are being increasingly explored for their capacity to selectively modify cancer-related transcripts and epitranscriptomic marks like m^6^A, showing great potential in precision oncology (Shi and Wu 2024; Xu et al. 2025). Unlike classical CRISPR systems that target DNA, Cas13 specifically recognizes and cleaves single-stranded RNA, making it particularly suitable for epitranscriptomic manipulation (Wang et al. 2023; Aloliqi et al. 2025). In experimental models, RNA-targeting CRISPR technologies have demonstrated the ability to selectively modulate cancer-related transcripts in vivo, for example in prostate cancer, potentially minimizing damage to healthy tissues, compared to conventional cancer therapies (Xu et al. 2025). Such strategies may help overcome therapy resistance, by targeting m^6^A-dependent pathways involved in CSC survival, proliferation and drug response (Xu et al. 2025).

Targeted therapeutic strategies directed against m^6^A machinery are also emerging. Small molecule inhibitors of m^6^A regulators show considerable potential for sensitizing resistant cancer cells to conventional therapies, including chemotherapy, radiotherapy and potentially immunotherapy (Su et al. 2020; Zhou et al. 2025; Shpiliukova et al. 2025; Liu and Kan 2025; Jiang et al. 2026). Among the most studied inhibitors, FTO-targeting compounds and ALKBH5 inhibitors have demonstrated anti-tumor activity in preclinical models, including suppression of leukemia and glioblastoma growth and reduction of CSC-like properties (Huff et al. 2021). Similarly, METTL3 inhibitors such as STM2457 and its derivatives have shown strong anti-leukemic effects by impairing translation of oncogenic transcripts and reducing leukemia stem cell self-renewal (Yankova et al. 2021). METTL3 inhibition has been shown to enhance tumour immunogenicity and sustain T-cell function, by reversing T-cell exhaustion, and promoting IFNγ and GzmB expression, thereby improving cytotoxicity responses during anti-PD-1 therapy (Wu et al. 2024). In addition, the YL-5092 small molecule blocked the binding of YTHDC1 to its m^6^A substrates and reduced mRNA stability, resulting in apoptosis and myeloid differentiation in AML cells. Notably, it functionally impaired leukemia stem cells while sparing normal hematopoietic counterparts (Zhao et al. 2026). Despite these advances, m^6^A-targeted therapies remain largely at the preclinical stage, and several challenges still limit their clinical translation, as m^6^A regulators often exhibit context-dependent functions in cancer while also participating in essential biological processes, raising concerns regarding collateral toxicity in normal stem cells (Liu and Kan 2025).

## Challenges and future directions

The field has advanced considerably in recent years; however, several critical challenges still hinder both the understanding of m^6^A biology in cancer and its translation into clinical applications. These challenges are particularly pronounced in the context of CSCs, which typically constitute a rare tumor subpopulation, are difficult to isolate and display substantial phenotypic plasticity (Batlle and Clevers 2017). Current evidence indicates that m^6^A modifications and their regulatory machinery can either promote or suppress tumorigenesis depending on the tumor type and biological context. Overall, studies across multiple tumor models support an association between m^6^A regulators and key CSC characteristics, including pluripotency, tumorigenicity, self-renewal and therapy resistance. Nevertheless, context dependency has been observed, resulting in opposing biological effects, even within the same tumor type (Shpiliukova et al. 2025), further complicating the interpretation of experimental findings and the translational value. Another major challenge arises from the complexity of the m^6^A machinery itself. Beyond the canonical “writers,” “readers” and “erasers,” these regulatory proteins belong to multigene families whose specificity, redundancy and context-dependent functions are not yet fully understood (Zaccara et al. 2019). Technical limitations also restrict progress in the field. Accurate mapping of m^6^A modifications in rare CSC populations remains challenging because conventional approaches often require large amounts of input RNA and may not adequately capture intratumoral heterogeneity. Emerging high-resolution approaches, including single-cell and spatial epitranscriptomics, are expected to address some of these limitations by enabling a more comprehensive characterization of m^6^A dynamics within heterogeneous tumor ecosystems. Such advances will likely be essential for the development of personalized therapeutic strategies based on the specific m^6^A modification landscapes of individual tumors.

From a therapeutic perspective, the development of effective small-molecule inhibitors targeting m^6^A regulators in CSCs remains difficult, particularly because of the need to achieve sufficient specificity while minimizing systemic toxicity. Recent studies suggest that combining m^6^A-targeted therapies with conventional anticancer treatments may help overcome therapeutic resistance, especially when coupled with the development of more selective inhibitors. Safety considerations are also paramount, emphasizing the need to preferentially target cancer-specific m^6^A regulators while sparing normal tissues (Uddin et al. 2024). Finally, the extensive crosstalk between m^6^A regulatory networks and other epigenetic and signaling pathways adds an additional layer of complexity to the clinical translation of these findings. A deeper understanding of the interplay between m^6^A modifications and CSC biology will therefore be crucial for the safe and effective implementation of m^6^A-based therapeutic strategies in oncology.

## Data Availability

No datasets were generated or analysed during the current study.
